# Forced virus evolution reveals functional crosstalk between the disulfide bonded region and membrane proximal ectodomain region of HIV-1 gp41

**DOI:** 10.1186/1742-4690-10-44

**Published:** 2013-04-23

**Authors:** Ashraf I Khasawneh, Annemarie Laumaea, David N Harrison, Anna K Bellamy-McIntyre, Heidi E Drummer, Pantelis Poumbourios

**Affiliations:** 1 Virus Fusion Laboratory, Burnet Institute, Prahran, VIC, 3004, Australia; 2 Department of Microbiology, Monash University, Clayton, VIC, 3168, Australia; 3 Department of Microbiology and Immunology, The University of Melbourne, Melbourne, VIC, 3010, Australia; 4 Present address: Department of Biomedical Science, Faculty of Medicine, The Hashemite University, Zarqa, 13115, Jordan

## Abstract

**Background:**

The disulfide-bonded region (DSR) of HIV-1 gp41 mediates association with gp120 and plays a role in transmission of receptor-induced conformational changes in gp120 to gp41 that activate membrane fusion function. In this study, forced viral evolution of a DSR mutant that sheds gp120 was employed to identify domains within gp120-gp41 that are functionally linked to the glycoprotein association site.

**Results:**

The HIV-1_AD8_ mutant, W596L/K601D, was serially passaged in U87.CD4.CCR5 cells until replication was restored. Whereas the W596L mutation persisted throughout the cultures, a D601H pseudoreversion in the DSR partially restored cell-free virus infectivity and virion gp120-gp41 association, with further improvements to cell-free virus infectivity following a 2nd-site D674E mutation in the membrane-proximal external region (MPER) of gp41. In an independent culture, D601H appeared with a deletion in V4 (Thr-394-Trp-395) and a D674N substitution in the MPER, however this MPER mutation was inhibitory to W596L/K601H cell-free virus infectivity. While cell-free virus infectivity was not fully restored for the revertant genotypes, their cell-to-cell transmission approached the levels observed for WT. Interestingly, the functional boost associated with the addition of D674E to W596L/K601H was not observed for cell-cell fusion where the cell-surface expressed glycoproteins function independently of virion assembly. The W596L/K601H and W596L/K601H/D674E viruses exhibited greater sensitivity to neutralization by the broadly reactive MPER directed monoclonal antibodies, 2F5 and 4E10, indicating that the reverting mutations increase the availability of conserved neutralization epitopes in the MPER.

**Conclusions:**

The data indicate for the first time that functional crosstalk between the DSR and MPER operates in the context of assembled virions, with the Leu-596-His-601-Glu-674 combination optimizing viral spread via the cell-to-cell route. Our data also indicate that changes in the gp120-gp41 association site may increase the exposure of conserved MPER neutralization epitopes in virus.

## Background

The entry of HIV-1 into cells follows receptor binding by the trimeric surface-exposed gp120 glycoprotein, which activates the membrane fusion function of the trimeric transmembrane glycoprotein, gp41. A globular head corresponding to the gp120 trimer encompasses much of the gp41 ectodomain [1,2], the association between gp120 and gp41 apparently trapping the glycoprotein complex in an energetically strained or “metastable” state. The sequential binding of gp120 to CD4 and then CCR5 or CXCR4 releases the trap, triggering the refolding of gp41 into a 6-helix bundle, which mediates membrane fusion and viral entry (see [3,4]). Membrane fusion involves insertion of the gp41 fusion peptide into the outer leaflet of the target membrane and the gp41 ectodomain adopting a prehairpin intermediate conformation that bridges the viral and cellular membranes [5-7]. 6-helix bundle formation brings together the N- and C-terminal membrane-inserted ends of gp41 (the fusion peptide and transmembrane domain), apposing the associated viral and cellular membranes for merger [8-10].

**Figure 1:**
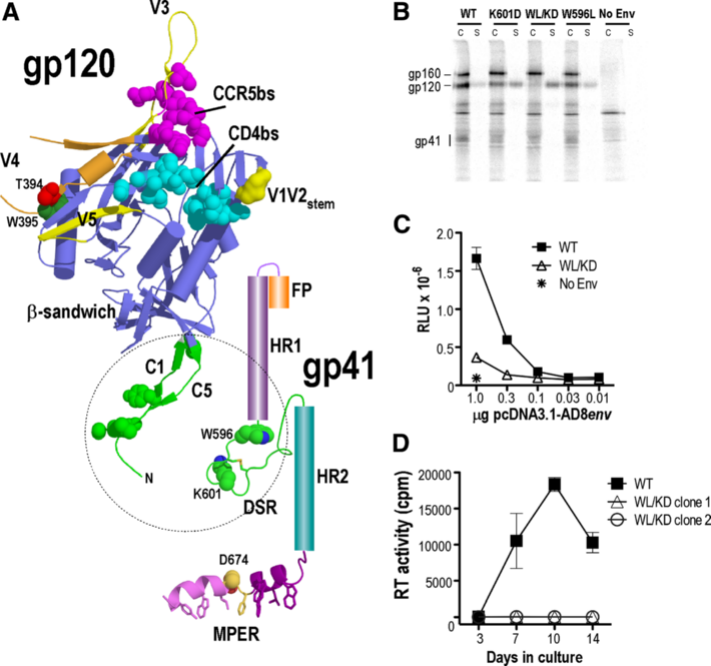
**Location and phenotype of WL/KD.** (**A**) Location of WL/KD in the context of the gp120-gp41 ectodomain. gp120 was drawn using the coordinates 3JWD [17] and 2QAD [20]. The gp120 core is colored blue, CD4 binding site (CD4bs) and CCR5-binding site (CCR5bs) in cyan and magenta, respectively, gp41 binding site in green. gp41: the DSR (green) and MPER were drawn using the coordinates 1IM7 [21] and 2PV6 [22]. The N- and C-terminal helical segments of the MPER are colored purple and magenta respectively and the interhelical hinge in yellow. The sidechains of the aromatic/hydrophobic face of the MPER that inserts into the lipid phase of the membrane are indicated. FP: fusion peptide. (**B**) gp120-gp41 association. Lysates of metabolically labeled WT, K601D, WL/KD, W596L or empty vector (No Env) transfected 293T cells (c) and corresponding culture supernatants (s) were immunoprecipitated with pooled IgG from HIV-1-infected persons and protein G-Sepharose. Proteins were analysed by reducing SDS-PAGE and phosphorimaging. (**C**) Cell-cell fusion activity. 293T effector cells were cotransfected with pCAG-T7 plus pcDNA3.1-AD8*env* plasmids and then cocultured (18 h, 37°C) with CD4 plus CCR5-expressing BHK21 cells harboring a luciferase reporter plasmid. The mean relative light units (RLU) of a representative experiment are shown. (**D**) 14-day replication kinetics in U87.CD4.CCR5 cells. Virus produced in 293T cells was normalised for RT activity and used to infect U87.CD4.CCR5 cells. The RT activity of the culture supernatant was measured at days 3, 7, 10 and 14, postinfection. The mean RT activity ± standard deviation of triplicate samples is shown.

Evidence is accumulating to suggest that the association site formed by the DSR of gp41 and the terminal conserved regions 1 (C1) and 5 (C5) of gp120 [11-13] act as a synapse for gp120-to-gp41 conformational signaling (Figure 1A). For example, the simultaneous introduction of Cys residues to the DSR and to C5 covalently links the gp41-gp120 heterodimer, trapping it in a fusion-inactive state with reduction of the intersubunit disulfide required to activate membrane fusion [14,15]. Furthermore, mutations in the DSR can uncouple CD4-gp120 binding from induction of the gp41 prehairpin intermediate, and can block the initial lipid-mixing or hemifusion phase of the membrane fusion cascade. These findings led to the proposal that the DSR acts as a sensor of receptor-induced conformational changes in gp120 leading to the fusion activation of gp41 [16]. A 7-stranded β-sandwich connecting the gp41-interactive C1 and C5 termini to the inner and outer domains of gp120 [17] also plays a role in mediating association with gp41 [18] and in regulating its activation state [19]. The β-sandwich links together 3 structurally plastic layers that are remodelled by CD4 engagement and coordinates the transmission of this conformational change to the gp41 association site, releasing gp41 from the metastable state.

The MPER, a highly conserved 23-residue Trp-rich sequence that connects the helical region 2 (HR2) of the gp41 ectodomain to the transmembrane domain, plays an important role in the membrane fusion mechanism [23-26]. This sequence has also been implicated in modulating receptor-induced changes in the gp120-gp41 complex, since simultaneous mutations in the MPER and the fusion-peptide proximal segment of gp41 block CD4-induced shedding of gp120 from the gp120-gp41 complex and the initial, lipid mixing phase of the membrane fusion cascade [27]. Spectroscopic studies of synthetic MPER peptides indicate that this segment is likely to reside in the lipid-polar head group interfacial region of the envelope, lying roughly parallel to the membrane plane, as a kinked helix comprising a tilted N-terminal helix (res. 664–672) connected by a short hinge to a C-terminal helix (res. 675–683) [22]. In current models, the MPER is associated with the virion envelope at the base of the metastable gp120-gp41 trimer, whereas following receptor activation, it associates with the fusion-peptide proximal segment to form a clasp that stabilizes the membrane-interactive end of the 6-helix bundle to enable the initiation of membrane fusion [28-31]. Three of the most broadly reactive neutralizing antibodies (brNAb) against HIV-1 (2F5, 4E10 and Z13) bind to the MPER and have been shown to contribute to protection against viral challenge of passively immunized macaques (see [23,24,32]). Thus the structure, function and immunogenicity of the MPER have been a focus of intense study for many years, however the design of vaccines that elicit 2F5- and 4E10-like antibodies has proven difficult (e.g. [33,34]).

The aim of this study was to identify determinants that are functionally linked to the “activation synapse”. To this end, we forced the evolution of a DSR mutant virus with disrupted gp120 association, HIV-1_AD8_-W596L/K601D (WL/KD), by sequential passage in U87.CD4.CCR5 cells. A D601H pseudoreversion in the DSR operated in conjunction with D674E in the MPER to improve gp120-gp41 association and to enable efficient viral spread in culture. The revertant W596L/K601H (WL/KH) and W596L/K61H/D674E (WL/KH/DE) viruses exhibited increased sensitivity to the broadly neutralizing MPER-directed MAbs 2F5 and 4E10. The data indicate that the MPER is functionally linked to the association/activation synapse of gp120-gp41 and suggest that changes in the gp120-gp41 association site may increase the exposure of conserved MPER neutralization epitopes in virus.

## Results

### Phenotype of WL/KD

The gp120-gp41 association phenotype of the WL/KD mutant was investigated by immunoprecipitation of biosynthetically labelled Env glycoproteins expressed in 293T cells [35]. The WL/KD mutation led to > 95% of total gp120 being sloughed into the culture supernatant (Figure 1B) indicating a shedding phenotype that was more severe than those of the component single K601D and W596L mutants. The loss of gp120-gp41 association for WL/KD corresponded with the inhibition of cell-cell fusion function in a luciferase reporter assay employing Env-293T effector cells and CD4 and CCR5-expressing BHK21 targets (Figure 1C). Consistent with these gp120-shedding and fusion defects, WL/KD blocked HIV-1_AD8_ viral replication in U87.CD4.CCR5 cells (Figure 1D).

### Long-term culture of HIV-1_AD8_-WL/KD

**Figure 2:**
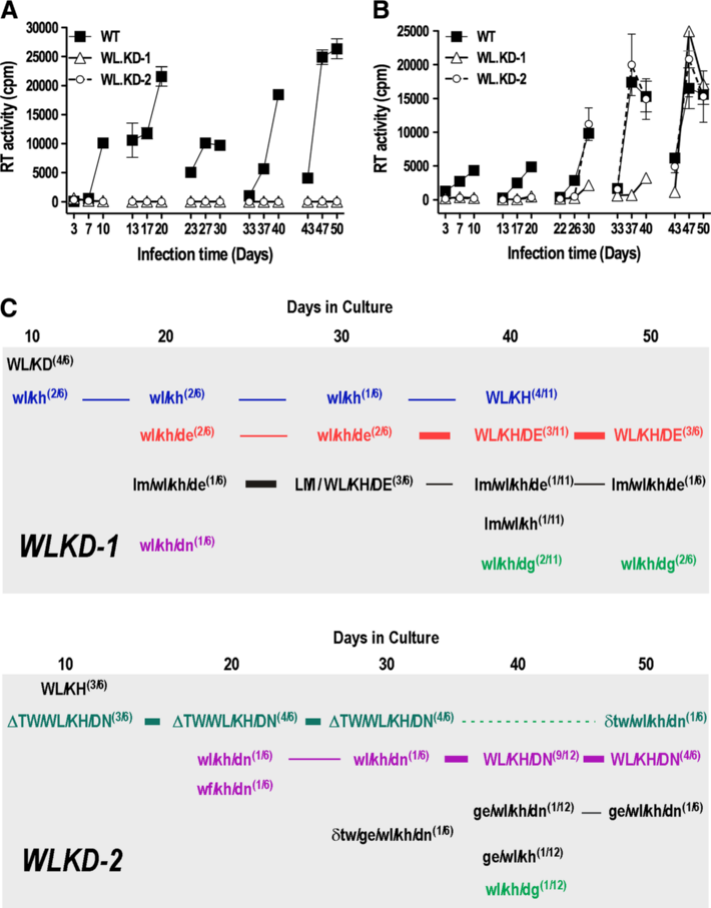
**Long-term culture of W596L/K601D virus.** (**A**) Wild type and W596L/K601D-mutated HIV-1_AD8_ virus stocks produced by transfected 293T cells were normalized according to RT activity and used to infect U87.CD4.CCR5 cells. The cell-free virus obtained at day 10 was filtered through a 0.45 μm nitrocellulose filter, normalized according to RT activity and used to infect fresh U87.CD4.CCR5 cells. Viruses were subjected to 5 sequential passages in total. (**B**) Infection of U87.CD4.CCR5 cells was initiated with VSV G-pseudotyped WT and W596L/K601D mutant viruses. The cells were trypsinized 24-h later to remove residually adsorbed viruses. The passaging procedure described in A was then followed. The results shown represent the mean RT activity ± standard deviation of triplicate samples. (**C**) Reversion pathways in WLKD-1 and WLKD-2 cultures. The *env* region was PCR amplified from proviral DNA isolated at days 10, 20, 30, 40 and 50, cloned into pΔKAD8*env* and sequenced. Upper case lettering connected by a bold horizontal line denotes a major evolutionary pathway, while lower case lettering connected via thin horizontal lines denotes a minor pathway. Lower case lettering only: low-frequency genotypes arising at the specified days.

Viruses derived from 2 independent HIV-1_AD8_ proviral clones carrying WL/KD were subjected to long-term culture in U87.CD4.CCR5 cells with serial passaging of cell-free virus onto fresh cells every 10 days. Evidence of replication was not observed for either clone, even after 50 days of culture (Figure 2A). The markedly diminished gp120-anchoring ability of WL/KD gp41 was assumed to have blocked viral entry and therefore reverse transcription, which is required for the generation of suppressor mutations. Mutant WL/KD HIV-1 particles were therefore pseudotyped with vesicular stomatitis virus glycoprotein G (VSV G) *in trans* in order to initiate HIV-1 envelope glycoprotein (Env)-independent infection via the endosomal pathway. Twenty-four h after infection, the U87.CD4.CCR5 cells were extensively washed and trypsinized to remove residual adsorbed virus prior to further culture for 10 days. The sequential passaging of the resultant cell-free virus in U87.CD4.CCR5 cells led to restored infectivity after 47 and 30 days in WL/KD cultures 1 and 2 (WLKD-1 and WLKD-2), respectively (Figure 2B).

The *env* region was PCR-amplified from genomic DNA isolated at days 10, 20, 30, 40 and 50, the PCR products were cloned into pΔKAD8*env*, and the entire *env* region was sequenced. *WLKD-1*. A D601H pseudoreversion emerged at day 10 (2/6 clones, WL/KH) prior to the appearance of D674E in the MPER at day 20, which persisted throughout the culture period. The genotypes observed over the 50-day culture period included WL/KH (9/35 clones), W596L/K601H/D674E (WL/KH/DE [10/35 clones]), L85M/W596L/K601H/D674E (LM/WL/KH/DE [6/35 clones]), W596L/K601H/D674G (WL/KH/DG [4/35 clones]), L85M/W596L/K601H (LM/WL/KH [1/35 clones]), and W596L/K601H/D674N (WL/KH/DN [1/35 clones]) (Figure 2C). *WLKD-2*. At day 10, 3/6 clones contained WL/KH, while 3 others contained the Thr-394-Trp-395 deletion in V4 (ΔTW), together with W596L and D601H in the DSR, and D674N in the MPER (ΔTW/WL/KH/DN). The ΔTW/WL/KH/DN genotype persisted to day 30 but at days 40 and 50, the dominant genotype was WL/KH/DN (13/18 clones) (Figure 2C). The W596L mutation was retained in 70/71 *env* clones obtained from the WLKD-1 and WLKD-2 cultures indicating a strong selection pressure to maintain Leu at 596. The K601H and D674E mutations were not observed in *env* clones obtained following passaging of the WT virus, while D674N was observed in 1 clone (data not shown).

### Infectivity of WL/KD revertants

**Figure 3:**
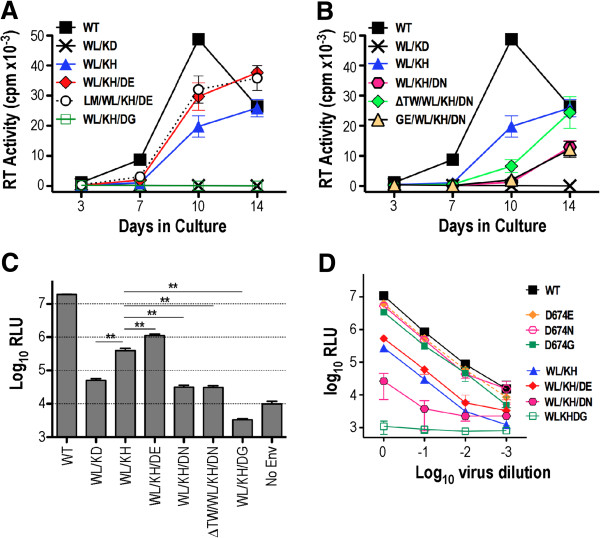
**Replication of representative WLKD-1 (A) and WLKD-2 (B) clones in U87.CD4.CCR5 cells.** Viruses produced in 293T cells were normalized according to RT activity and used to infect U87.CD4.CCR5 cells. Reverse transcriptase activity in culture supernatants was measured at days 3, 7, 10 and 14. The mean RT activity ± standard deviation of triplicate samples is shown. (**C**) Single-cycle infectivity was determined in U87.CD4.CCR5 cells infected with Env-pseudotyped luciferase reporter viruses at 48-h postinfection. Luciferase activity was normalized against the RT activity present in each virus inoculum. The mean RLU ± standard errors of 3 independent assays are presented here. **, *P* < 0.01, unpaired t test assuming unequal variances. (**D**) Serial 10-fold dilutions of Env-pseudotyped luciferase reporter viruses were added to U87.CD4.CCR5 target cells and luciferase activity determined 48 h later. The mean RLU ± standard deviations from a representative experiment are presented.

The dominant genotypes were reconstructed in the context of the pAD8 proviral clone. In the case of WLKD-1, cell-free virus-initiated replication in U87.CD4.CCR5 cells was partially restored by D601H in the DSR (WL/KH) and was optimised further by D674E in the MPER (WL/KH/DE) (Figure 3A). The addition of L85M to WL/KH/DE did not improve replication any further. Interestingly, the WL/KH/DG combination was replication-incompetent. Gly-674 can arise via an A-to-G mutation in the 2nd position of the Asp and Glu codons but in a WL/KH context appears to be an evolutionary dead-end. These data suggest that D601H and D674E can act synergistically to suppress the original replication defect. For WLKD-2, step-wise improvements in replication competence were observed with WL/KH/DN, ΔTW/WL/KH/DN and WL/KH, respectively (Figure 3B). Thus D674N is inhibitory to cell-free virus initiated replication in the context of WL/KH with ΔTW partially relieving this inhibition. The G145E V1 mutation observed at days 30–40 did not confer a replication advantage to WL/KH/DN. Interestingly, the replication competence of WLKD-2 genotypes were inferior to those derived from the WLKD-1 culture even though revertant virus emerged in the WLKD-2 culture first, suggesting that additional mechanisms of reversion were operating in this culture.

The infectivity associated with revertant genotypes was further examined in a single cycle infectivity assay employing Env-pseudotyped luciferase reporter viruses. The infectivity of WL/KD for U87.CD4.CCR5 cellular targets was reduced by ~ 2.5log_10_ with respect to WT (Figure 3C). The D601H pseudoreversion in WL/KH increased this infectivity by ~10-fold while the addition of D674E led to a further 2-fold improvement, but the entry competence of WL/KH/DE remained 20-fold lower than WT. The addition of D674G to WL/KH (WL/KH/DG) markedly suppressed viral entry consistent with the observed lack of replication. The alternate MPER mutation, D674N, was inhibitory on the WL/KH background, consistent with the relative replicative capacity of WL/KH/DN and WL/KH, while the addition of ΔTW to WL/KH/DN did not improve single-cycle entry competence any further.

We next determined if the modulation of infectivity by D674E, D674N and D674G occurred via a functional link to Leu-596 and His-601 or whether it could be explained by a generalized enhancement or inhibition in Env function. Figure 3D indicates that the D674 mutations did not alter the infectivity of Env-pseudotyped luciferase reporter virus when introduced to the WT background, indicating a specific functional interaction between Leu-596, His-601 in the DSR and position 674 in the MPER.

### Cell-cell spread of revertant viruses

**Figure 4:**
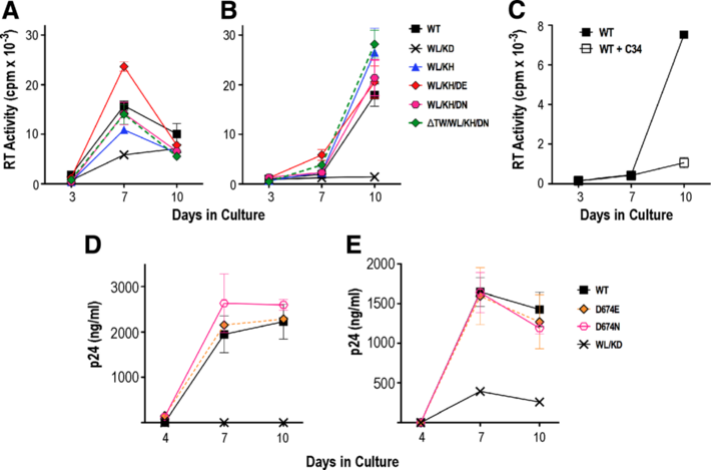
**Spread of cell-associated virus.** U87.CD4.CCR5 cells were inoculated with VSV G-pseudotyped HIV-1 particles [50,000 (**A**) or 20,000 (**B**) cpm of RT activity per inoculum] and then trypsinized 24-h later to remove residual adsorbed virus. The cells were then replated and cultured for a further 10 days. The results shown represent the mean RT activity ± standard deviation of triplicate samples. (**C**) As for B except that 1 μM C34 peptide was maintained in the culture following the trypsinization step. Effects of D674E and D674N mutations on viral spread initiated by cell-free (**D**) and cell-associated (**E**) virus in 10-day U87.CD4.CCR5 cultures. The results shown represent the mean p24 content ± standard deviation of duplicate samples obtained from the cultures.

The functional advantages conferred by D601H and D674E were less obvious in the single cycle infectivity assay when compared to 14-day replication experiments (compare Figure 3A, B and C). This apparent discrepancy may be explained by the fact that only a single cycle of infection mediated by cell-free virus occurs in the reporter assay, whereas multiple rounds of infection mediated by both cell-free and cell-associated virus occur in the replication assay [36,37]. We therefore inoculated U87.CD4.CCR5 cells with HIV-1-VSV G pseudotyped particles, reasoning that the highly fusogenic nature of VSV G will normalize the cellular entry of cell-free WT and revertant viruses in the first 24 hours of infection, thereby enabling an assessment of virus production following multiple rounds of cell-cell and cell-free viral transmission. At 24-h postinfection, the cells were trypsinized to remove residual surface-adsorbed virus, replated and then cultured for a further 10 days. Virus production was elevated for WL/KH/DE with respect to WT at day 7 and approached WT levels at day 10, whereas WL/KH, WL/KH/DN and ΔTW/WL/KH/DN replication was almost identical to WT over the 10-day culture (Figure 4A). A low level of reverse transcriptase (RT) activity was observed for WL/KD, which is likely due to a combination of virus production by cells infected by VSV G-pseudotypes in the initial 24 h plus low-level cell-cell spread. The results were confirmed in an experiment employing smaller inocula (20,000 cpm RT activity-equivalents of VSV G-HIV-1 pseudotypes) but virus production was delayed to day 10 in this case (Figure 4B). Notably, the presence of D674N was not inhibitory to replication when combined with WL/KH in this infection system. We found that viral spread in VSV G-HIV-1 pseudotype initiated cultures was blocked by the C34 fusion inhibitor peptide, consistent with viral spread being HIV-1 Env dependent (Figure 4C). These data suggest that the D601H pseudoreversion and 2nd (and 3rd) site mutations optimise viral spread mediated by cell-associated virus.

We next determined whether D674E and D674N on a WT HIV-1_AD8_ background had any effect on cell-free or cell-associated viral transmission in 10-day U87.CD4.CCR5 cultures. The cells were inoculated with 50,000 cpm RT-equivalents of non-pseudotyped (Figure 4D) or VSV G-pseudotyped (Figure 4E) virions. At 24-h postinfection, the cells inoculated with VSV G pseudotypes were trypsinized and replated. The virus content of the culture supernatants at days 4, 7 and 10 postinfection was quantified by p24 ELISA. The data show that D674E and D674N did not affect cell-free or cell-cell viral transmission (Figure 4D and E, respectively). By contrast, WL/KD blocked virus transmission in both cases. These data further support the conclusion that position 674 in the MPER is specifically linked to Leu-596 and His-601 in the DSR.

### Glycoprotein expression and subunit association

**Figure 5:**
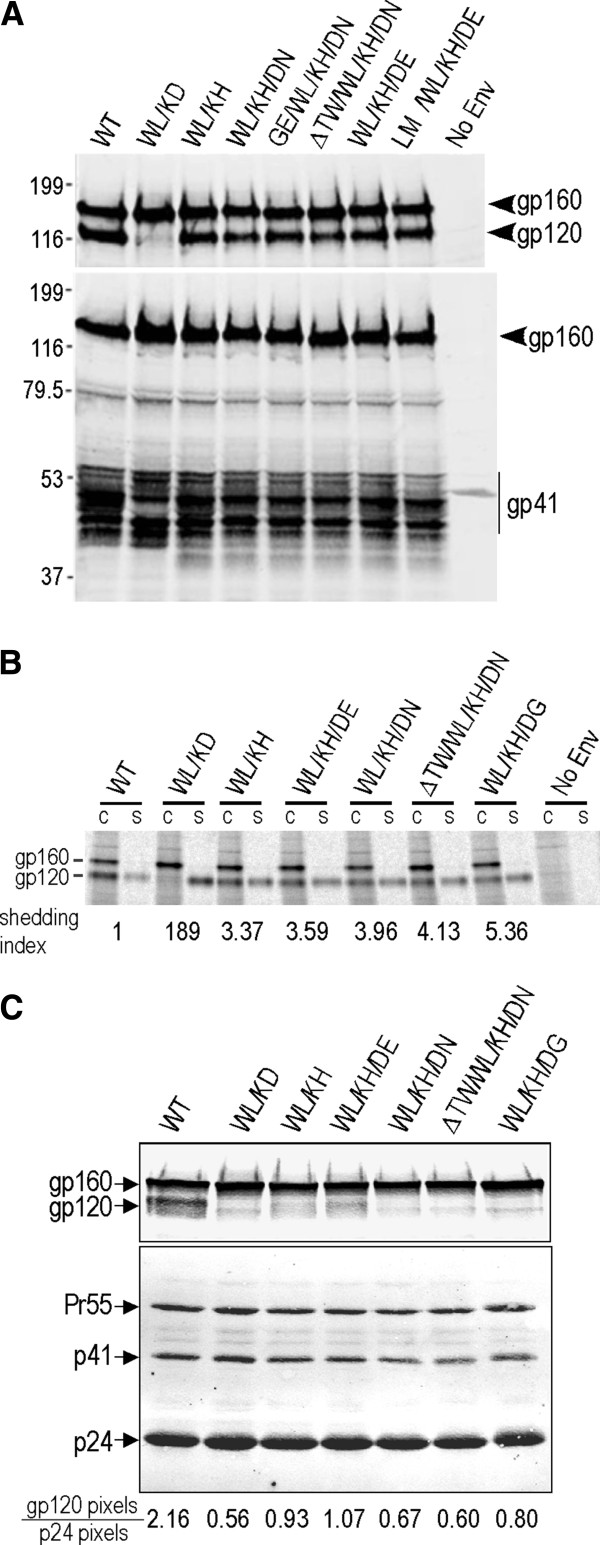
**Biochemical analysis of selected revertant clones.** (**A**) Western blotting. At 48-h posttransfection, pΔKAD*env*–transfected 293T cells were lysed and subjected to reducing SDS-PAGE followed by western blotting with DV-012 to gp120 (upper panel) and mAb C8 to gp41 (lower panel). (**B**) gp120-gp41 association was determined as for Figure 1B. gp120-shedding index was calculated according to the formula: ([mutant gp120]_supernatant_ x [WT gp120]_cell_)/([mutant gp120]_cell_ x [WT gp120]_supernatant_) [12]. (**C**) Characterization of virions produced by pAD8 infectious clones. Pelleted HIV-1 virions were analysed by Western blotting using DV-012 (upper panel) and pooled IgG from HIV-1-infected persons (lower panel). gp120 and p24 band intensities were determined using a Licor Odyssey scanner.

The synthesis and processing of the cloned Env glycoproteins were examined by western blot. The gp120-specific polyclonal antibody, DV-012, (Figure 5A, upper) revealed that similar levels of gp160 were expressed for all clones and that cell-associated gp120 was present for WT and His-601-containing clones. Consistent with the shedding defect seen previously (Figure 1B), gp120 was largely absent for WL/KD. The monoclonal antibody (mAb) C8, directed to a linear epitope within the cytoplasmic domain of gp41 [38], revealed similar levels of gp160 and gp41 expression for the Env constructs examined (Figure 5A, lower). Interestingly, the presence of the WL/KD mutation in gp41 resulted in a distinct glycosylation pattern relative to the other clones, suggesting a subtly different structure. The gp120 anchoring ability of the cell-surface expressed revertant Env proteins was analyzed by immunoprecipitation of pulse-chase biosynthetically labelled Env transfected 293T cells. Figure 5B again confirms the gp120 shedding defect of WL/KD and indicates that the subsequent D601H mutation, present in the WL/KH clone was sufficient to partially restore gp120 association levels with no further improvements to association following the addition of 2nd and 3rd site mutations. By contrast, Western blot analysis of viruses pelleted from the supernatants of 293T cells transfected with pAD8 proviral clones revealed gp120 shedding phenotypes for the revertants (Figure 5C). These data indicate that stable gp120-gp41 association is not conferred by the reverting mutations in a cell-free virion context. The apparent discrepancy between cellular and virion Env could be related to the fact that cellular Env was analysed 5 h post synthesis whereas virions were analysed at 48 h posttransfection. It may be that a subtly unstable gp120-gp41 complex becomes more evident over time. Interactions between the cytoplasmic tail of gp41 and the MA domain of Gag in the context of cell free virions have been found to impose structural and functional constraints on the Env ectodomain [39-42]. These data suggest an alternative scenario where WL/KH-containing revertant Envs attain apparently WT gp120-gp41 stability when expressed in the absence of other viral proteins, whereas the corresponding virion-associated glycoprotein complexes are less stable in an Env conformation that is modulated by internal Env-Gag interactions.

### Examination of the 601–674 functional linkage in cell-cell fusion

**Figure 6:**
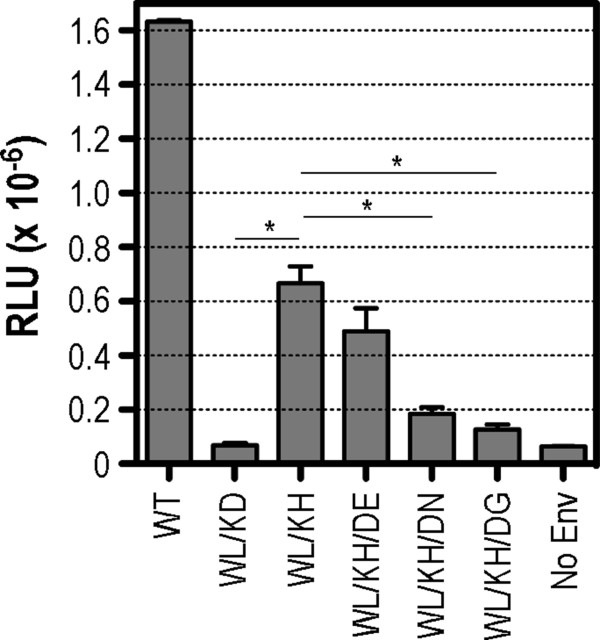
**Cell-cell fusion activities of revertant Envs.** 293T effectors (cotransfected with pΔKAD8*env* plus pCAG-T7) were cocultured with BHK-21 targets cotransfected with pT4luc and pcCCR5 vectors for 18 h and then lysed and assayed for luciferase activity. The data presented here are the means ± standard errors of 3 independent assays. *, *P* < 0.05, unpaired t test assuming unequal variances.

We next examined the membrane fusion activities of selected revertant Env sequences in a cell-to-cell fusion assay. In this context, Env is expressed in the absence of other viral proteins and is therefore not subjected to the conformational constraints that may be imposed by matrix-gp41 cytoplasmic tail interactions present in virus [39-42]. The assay was conducted at limiting Env concentrations (0.25 μg pΔKAD*env*) to enable detection of subtle changes in fusion function. Consistent with the cell-free virus infectivity data, WL/KD blocked cell-cell fusion, WL/KH exhibited partially restored fusion function and D674N and D674G mutations were inhibitory in a WL/KH context (Figure 6). However, in contrast to the infectivity data, D674E did not enhance fusogenicity when added to WL/KH. These data suggest that the functional interaction between Leu-596, His-601 and Glu-674 largely operates in the context of assembled virions transmitted via the cell-cell route and the conformational constraints imposed by Gag-gp41 cytoplasmic tail interactions [39-42].

### Neutralization sensitivity of WL/KH and WL/KH/DE mutants

**Figure 7:**
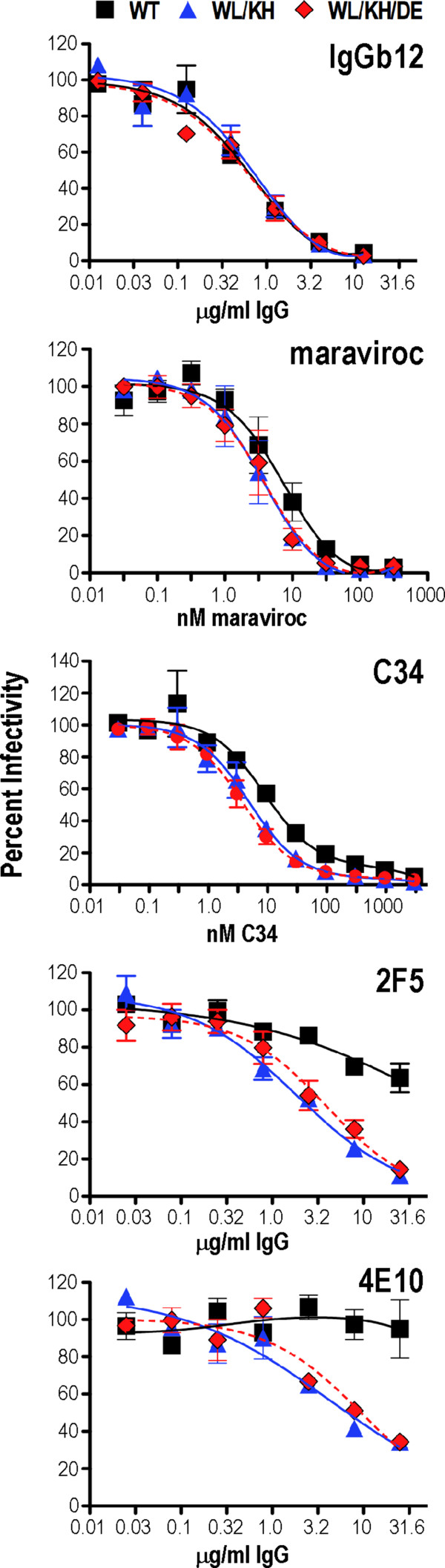
**Sensitivity of revertants to neutralizing agents.** The TZM-bl cells were incubated with virus-inhibitor complexes for 2 days prior to lysis and then assayed for luciferase activity. For the maraviroc experiment, target cells were incubated in the presence of the inhibitor for 1 h prior to inoculation. Neutralizing activities are reported as the average percent maximal luciferase activity. The data presented here are the means ± standard errors; n = 2 for IgGb12, 2F5 and 4E10; n = 3 for C34 and maraviroc.

To determine whether WL/KH and WL/KH/DE are associated with structural changes in gp41, neutralizing agents were used to probe functional virion-associated gp120-gp41 complexes. Virus stocks, produced by transfecting 293T cells with pAD8 infectious clones, were adjusted to produce ~ 1.5 × 10^6^ RLU following 48 h of infection of TZM-bl cells. The viruses were then incubated with the neutralizing agents for 1 h prior to infection of naïve TZM-bl cells. In the case of the CCR5 antagonist maraviroc, target cells were pretreated with the inhibitor for 1 h prior to infection. The CD4 binding site brNAb, IgGb12, and the CCR5 antagonist, maraviroc, neutralized WT, WL/KH and WL/KH/DE to similar extents (the maraviroc IC_50_ and IC_90_ values for WT were not significantly different to those obtained with the mutants) indicating that gp120-CD4-CCR5 interactions had not been affected by the mutations in gp41 (Figure 7). Small, but significant ~ 0.5log_10_-decreases in C34 IC_50_, a gp41 HR2 peptide analog that binds to the helical region 1 (HR1) coiled coil in a fusion intermediate conformation of gp41 [6,43,44], were observed for the revertants in relation to the WT (*P* < 0.05, WL/KH and WL/KH/DE versus WT; 2-tailed t test, unequal variances). Notably, WL/KH and WL/KH/DE exhibited markedly greater sensitivity to neutralization by the MPER-specific brNAbs 2F5 and 4E10 when compared to the WT. These data are consistent with structural changes in the gp120-gp41 complex that increase the availability of neutralization targets in the MPER of gp41.

## Discussion

The forced evolution of WL/KD mutant viruses with severely disrupted gp120-gp41 association led to the emergence of replication-competent revertants containing a D601H pseudoreversion in the DSR plus D674E or D674N 2nd site mutations in the MPER. In a second culture, revertant clones contained the ΔT394-W395 deletion in V4 in addition to D601H and D674N. The WL/KH and WL/KH/DE viruses exhibited greater sensitivity to the brNAbs, 2F5 and 4E10, indicating that the restoration of function was associated with structural changes in Env that increase the accessibility of neutralization epitopes within the MPER. Our data reveal a functional linkage between the DSR and MPER of gp41 and point to a novel approach for improving the accessibility of conserved neutralization epitopes within the MPER in a virion context.

The severe shedding phenotype of WL/KD is likely to have resulted from the combined effects of decreased hydrophobic sidechain bulk and an additional negative charge in the contact site, that destabilizes gp120-gp41 association. Phenotypic analysis of the revertant genotypes indicated that D601H is a key evolutionary step that partially restores gp120-gp41 association. Histidine at 601 introduces an imidazole moiety into the association site, which would partially compensate for the loss of the indole ring of Trp-596 and removes the negative charge contributed by Asp-601. Interestingly, Leu-596 was maintained in both long-term cultures, indicating that the smaller hydrophobic sidechain is preferred at this position when His is present at 601. While His is expected to be neutral at physiological pH, its imidazole ring may become protonated if it is proximal to acidic residues. In this case, the D601H mutation would also restore positive charge to position 601, which may also lend stability to the gp120-gp41 association site. The combination of His-601 with D674E led to improved single-cycle and multi-cycle cell-free virus-initiated infectivity without detectable further improvement in gp120-gp41 association. The MPER mutation therefore appears to act at the level of virus entry. Highly mutable viruses, such as HIV-1 have the capacity to overcome fitness defects via multiple evolutionary pathways. It was therefore notable that we found a functional link between positions 601 and 674 of gp41 in parallel cultures, indicating a reproducible genetic link between the DSR and MPER. However, we cannot rule out the possibility that additional solutions to restore the gp120-gp41 interaction would be revealed with more independent evolution cultures of WL/KD.

**Figure 8:**
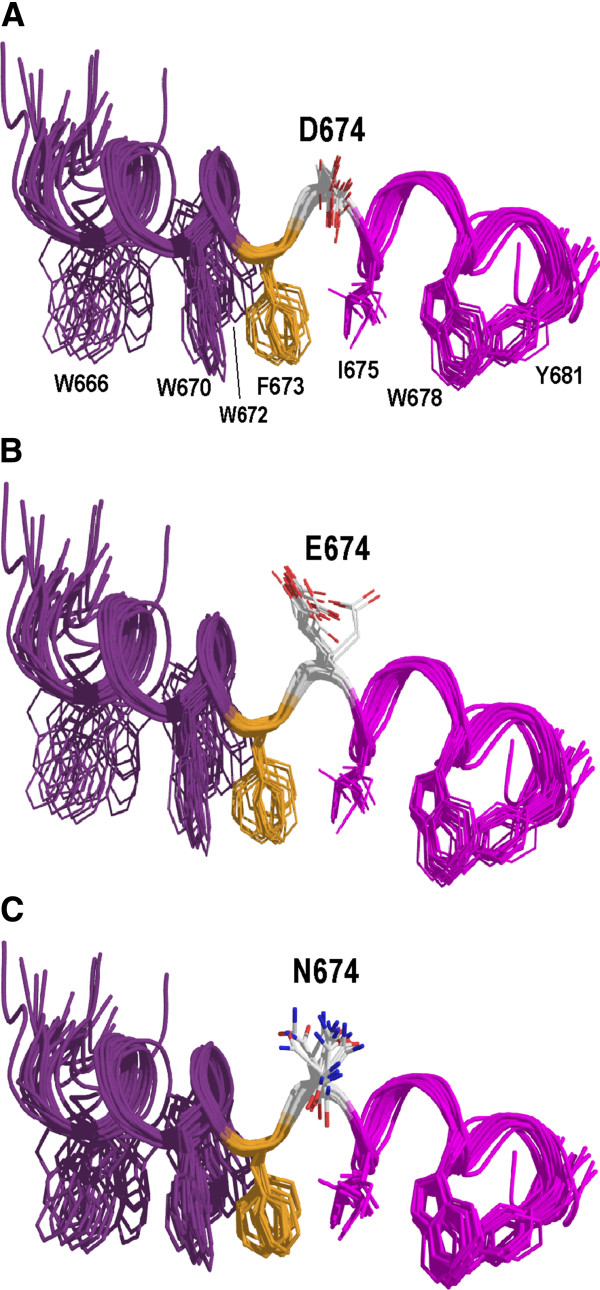
**Amino acid changes at position 674 modeled on the dodecylphosphocholine-associated MPER peptide (PDB entry 2PV6).** The Asp-674 (**A**), Glu-674 (**B**), and Asn-674 (**C**) models were produced with Swiss Model and drawn with Pymol. The N- and C-terminal helical segments are shown in purple and magenta respectively, while Phe-673 that forms part of the hinge region is in yellow. The aromatic layer and Ile-675, which are associated with the hydrophobic phase of the lipid are indicated. The N-terminus is to the left.

Nuclear magnetic resonance studies of synthetic MPER peptides suggest that in membranes, the MPER is a metastable L-shaped structure comprised of N- and C-terminal helical segments that are connected via a hinge composed of Phe-673, which is buried in the lipid phase, and a polar residue at position 674, which is solvent-exposed (Figure 8) (PDB entry, 2PV6 [22]. The C-terminal helix is likely to interact with the transmembrane domain and cholesterol in the lipid phase via the Leu-679-Trp-Tyr-Ile-Lys cholesterol recruitment motif [45-48], while the N-terminal helix represents a more flexible segment that might be in a metastable state and contains 3 Trp residues that are critical for membrane fusion [25]. Molecular modeling predicts that the original Asp side chain at position 674 will hydrogen bond via Oδ1 with the backbone amides of Asn-677 and Ile-675 in 17 of 17 MPER conformers (Figure 8A), thereby conferring rigidity to the interhelical hinge. By contrast, an additional methyl group within the Glu-674 sidechain moves the terminal carboxylate out of hydrogen bonding range in 15/17 conformers, consistent with hinge flexibility (Figure 8B). The boost to the infectivity of WL/KH cell-free virus upon addition of D674E may therefore be related to increased MPER flexibility. Interestingly, the addition of D674E to W596L/K601H only marginally improved gp120-association for cell-free virions whereas single-cycle infectivity was improved some 20-fold. It may be that increased MPER hinge flexibility due to D674E relieves or corrects a structural constraint on WL/KH-containing Env to enable stronger gp120-gp41 association within the virion glycoprotein complex, thereby enhancing entry function. It is also possible that D674E directly enhances an MPER-related function in virus-cell fusion [25,49]. For example, greater flexibility in the MPER may facilitate its relocation from the lipid-polar head group interfacial region of the envelope for interaction with the fusion peptide-proximal segment during terminal clasp formation at the membrane-proximal end of the 6-helix bundle during the initiation of fusion [28,30]. Alternatively, the membrane perturbing properties of the MPER may be enhanced by D674E [26,50-52].

Mutational studies have indicated that the DSR is associated with the C1 and C5 regions of gp120 [11-13,35], which project from the base of gp120 [17] (Figure 1A). The modeling of these projections in a trimeric context suggests that they will form a layer that encases the DSR [16], thereby occluding it from antibody recognition [53,54]. On the other hand, the MPER is believed to occupy a spatially distinct location at the base of the gp41 trimer, partially embedded in the envelope and in certain HIV-1 strains, available for antibody binding [53-55]. The MPER-DSR functional linkage is therefore likely to operate via an allosteric mechanism that involves other structural elements of the ectodomain. The HR1 of gp41 is implicated as one such element by the finding that T569A (HR1) and I675V (MPER) polymorphisms synergize in conferring a neutralization sensitive Env conformation [56]. Furthermore, fusion inhibitor- and neutralizing antibody-driven viral evolution studies have suggested functional linkages between HR1, the receptor and coreceptor binding sites of gp120, and HR2 [57,58]. We did not observe significant changes in the sensitivity of cell-free viruses to the CD4-binding site-directed brNAb b12 or to maraviroc, suggesting that alterations to the receptor and coreceptor binding sites did not contribute to the mechanism of reversion. However, a small (0.5log_10_) but significant increase in C34 sensitivity was observed for WL/KH and WL/KH/DE viruses. Subtle alterations to the function of HR1 during fusion, such as prehairpin coiled coil formation, may accompany the WL/KH and WL/KH/DE changes.

In contrast to the data obtained with cell-free virions, replication levels at (WL/KH, WL/KH/DN, ΔTW/WL/KH/DN) or better (WL/KH/DE) than those of WT were achieved by the revertants when infections were initiated with VSV G pseudotyped viruses. In this latter system, the initial infection rounds will be largely mediated by the highly fusogenic VSV G present in the viral envelope thereby normalizing WT and mutant virus infectivity, while subsequent infection rounds will be mediated by gp120-gp41 present on virions transmitted directly from cell to cell via virological synapses in addition to nascent cell-free virions. Direct cell-cell viral transmission has been calculated to be at least 8-times more efficient than cell-free viral spread and is believed to be due to higher effective multiplicity of infection and virus viability within virological synapses [37]. It may be that in the case of cell-free virions, which encounter receptors following solution-phase diffusion, gp120 is shed from unstable WL/KH, WL/KH/DE and WL/KH/DN gp120-gp41 complexes during the lag time between budding and attachment, thereby decreasing infectivity over time. Consistent with this idea, gp120-gp41 association was observed to be relatively weak in cell-free revertant virions. By contrast, in directed viral transmission across virological synapses, virion budding, receptor binding, virion maturation and entry appear to be closely linked and to occur over short timeframes [36,59,60], which may limit the loss of gp120 from virions prior to receptor encounter. An alternative scenario is suggested by the recent work of Dale et al. [59] who found that in cell-cell viral transmission, receptor attachment is mediated by immature virions, and Env activation for fusion occurs later following viral maturation in the endosome. This contrasts cell-free virus infection where receptor binding is largely mediated by mature virions. It may be that the reverting mutations act optimally in the context of viral gp120-gp41 complexes that are initially maintained in an inactive form, through interactions between the gp41 cytoplasmic domain and immature Gag [40,42], prior to receptor engagement and activation for fusion. Overall, these data suggest that the cell-cell mode of viral spread plays the key role in the mechanism of reversion. This idea is consistent with the finding that the WL/KH/DN and ΔTW/WL/KH/DN *env* genotypes coexist with WL/KH in the WLKD-2 culture even though the former exhibited lower cell-free virus infectivity. It is interesting that the boosts to infectivity associated with the addition of D674E to W596L/K601H did not correlate with enhanced cell-cell fusion activity, where the cell-surface expressed glycoproteins function independently of other virion components. These data are consistent with a Leu-596-His-601-Glu-674 functional interaction that is dependent on virion assembly and the structural constraints imposed on the Env ectodomain, including the MPER, by Gag-gp41 cytoplasmic tail interactions [39-42].

The WL/KH (and WL/KH/DE) mutations were associated with increased sensitivity to the 2F5 and 4E10 MPER-directed brNAbs, indicating a structural change in Env that increases MPER accessibility. Two neutralization mechanisms have been proposed for 2F5 and 4E10: *i)* direct interaction with the MPER in neutralization-sensitive viral strains; *ii)* Env-receptor interaction-triggered MPER accessibility to brNAb in resistant strains [55]. We have found that the macrophage-adapted AD8 strain is relatively resistant to these brNAbs, suggesting that the MPER is sterically occluded in the WT viral AD8 Env complex. The WL/KH gp120-gp41 association site mutation may lead to a more open Env structure, enabling better epitope access for 2F5 and 4E10. Alternatively or additionally, WL/KH may be associated with structural change in the MPER itself, with increased MPER flexibility and/or altered membrane interactions facilitating paratope-mediated extraction of the 2F5 and 4E10 epitopes from the envelope. These proposed changes to the MPER may be present in cell-free virions or could occur during the virus-cell fusion process [55].

MPER-specific brNAbs are of particular interest to the HIV-1 vaccine field due to the conserved nature of their epitopes and their neutralization breadth [23,61-64]. Biophysical and structural studies have indicated that membrane-anchored MPER conformations are optimally bound by 2F5 and 4E10-like brNAbs [65], but the goal of developing a vaccine that presents the MPER in a lipid environment and produces high-titre 2F5- and 4E10-like antibodies with broad neutralization properties has not yet been realized [34,66-69]. This may in part be due to poor mimicry of MPER conformations in artificial lipopeptide immunogens that are required to elicit the appropriate antibody ancestor and to then drive a high degree of somatic mutation that gives rise to mature brNAbs, such as 4E10 [70,71]. Our finding that WL/KH in the gp120-gp41 association site can sensitize HIV-1 to 2F5 and 4E10 suggests that the MPER is more accessible to these brNAbs. It is plausible that incorporation of WL/KH (or WL/KH/DE) into a HIV-like particle immunogen may increase the exposure of MPER epitopes in a quasi-native context and in conformations that promote the production of 2F5/4E10-like specificities. Such an approach would also enable modifications to gp120, itself a major target of brNAbs and apparently protective vaccine responses [72-74], to improve its association with gp41 and its immunogenicity in regard to brNAbs. We consider that the approach used here may help researchers to identify mutations that would increase the stability and gp120 retention of Env trimer-based immunogens.

We previously reported that the contributions of Trp-596 and Lys-601 to gp120-gp41 association and membrane fusion are influenced by sequence changes in V1, V2, and V3 [35], which are predominantly associated with the evolution of neutralization resistance [75-81], as well as coreceptor preference and cellular tropism [82-85]. Thus the gp120-gp41 association site appears structurally and functionally adaptable, perhaps to maintain glycoprotein function during gp120-gp41 evolution. The observation here of functional crosstalk between the DSR and MPER implies that the structural adaptation of the gp120-gp41 synapse in order to cope with the evolution of other glycoprotein domains is also linked to changes in MPER structure that alters the ability of conserved neutralization epitopes therein to be bound by antibody.

## Conclusions

We have found, by using a forced virus evolution approach, evidence for functional crosstalk between the gp120-gp41 association site and the MPER. Our data indicate that looser gp120-gp41 association due to the WL/KH mutation within the DSR is linked to enhanced neutralization by brNAbs directed to the MPER. Our work points to a new approach for improving the accessibility of conserved MPER neutralization epitopes in virion-based immunogens.

## Methods

### Env expression vectors and proviral clones

The CMV promoter-driven HIV-1_AD8_ Env expression vector, pCDNA3.1-AD8*env*, is described elsewhere [35]. pΔKAD8*env* was derived by religation of the end-filled *Hind*III and *Eco*RI sites of pCDNA3.1-AD8*env*. Mutants of the pAD8 infectious clone (obtained from K. Peden [86]) were prepared by transferring the *Eco*RI-*Bsp*MI *env*-containing fragment from pCDNA3.1-AD8*env* vectors into pAD8. *In vitro* mutagenesis of the gp41 region was carried out using the Quikchange protocol (Stratagene).

### Infection of U87.CD4.CCR5 cells

Virus stocks were prepared by transfecting 293T cell monolayers with pAD8 infectious clones using Fugene 6 or Fugene HD (Roche). Virus-containing transfection supernatants were normalized according to RT activity, and then used to infect U87.CD4.CCR5 astroglioma cells (from H. Deng and D. Littman [87], NIH AIDS Research and Reference Reagent Program) in 25 cm^2^ culture flasks. The supernatants were assayed for RT activity [88] or p24 content (NCI p24 antigen capture ELISA) at various time points. To assess the transmission of cell-associated viruses, HIV-1 particles were pseudotyped with VSV G by cotransfection of 293T cells with pAD8 and pHEF-VSV G (from Dr. L.-J. Chang [89] NIH AIDS Research and Reference Reagent Program). U87.CD4.CCR5 cells in 25 cm^2^ culture flasks were inoculated with the HIV-VSV G pseudotypes, and then, at 24-h postinfection, trypsinized to remove surface-adsorbed virions. The cells were replated and then cultured for 10 days. The culture supernatants were assayed for RT activity or p24 content at various time points. For long-term cultures of viral mutants, the day-10 cell-free culture supernatants were filtered (0.45 μm pore size) and normalized according to RT activity prior to the next passage (5 passages in total). Genomic DNA was extracted from infected cells using Qiagen DNeasy Blood and Tissue kit. The viral DNA fragment encompassed by nucleotides 5954–9096 (HIV-1_HXB2R_ numbering convention) was PCR-amplified using Expand HiFi (Roche) and the primers, 5’-GGCTTAGGCATCTCCTATGGCAGGAAGAA (Env1A) and 5’-TAGCCCTTCCAGTCCCCCCTTTTCTTTTA (Env1M) [90]. The amplified sequences were ligated into pΔKAD8*env* (*Kpn*I-*Xba*I) and the entire *env* open reading frame sequenced using ABI BigDye terminator v3.1.

### Single cycle infectivity assays

Single cycle infectivity assays were conducted as described [27]. Env-pseudotyped luciferase reporter viruses were produced by cotransfecting 293T cells with pΔKAD8*env* plus the luciferase reporter virus vector, pNL4.3.Luc.R^-^E^-^ (NIH AIDS Research and Reference Reagent Program, from N. Landau [91]), using Fugene HD. The infectivity of pseudotyped viruses was determined in U87.CD4.CCR5 cells using the Promega luciferase assay system at 48 h postinfection.

### Western blotting

Twenty four h after transfection with pΔKAD8*env* vectors, 293T cells were lysed for 10 min on ice in PBS containing 1% Triton X-100, 0.02% sodium azide, 1 mM EDTA. The lysates were clarified by centrifugation for 10 min at 10,000 ×*g* at 4°C prior to SDS-PAGE under reducing conditions. The proteins were transferred to nitrocellulose and blotted with antibodies C8 to gp41 [38] and DV-012 to gp120 [92] (from G. Lewis and M. Phelan, respectively, NIH AIDS Research and Reference Reagent Program). The immunoblots were developed with Alexa Fluor 680-conjugated goat anti-mouse or donkey anti-sheep immunoglobulin (Invitrogen) and scanned in a LI-COR Odyssey infrared imager. For virion analysis, supernatants from pAD8-transfected 293T cells were centrifuged over 1.5 ml 25% w/v sucrose/PBS cushions (Beckman SW41 Ti rotor, 25,000 rpm, 2.5 h, 4°C) prior to reducing SDS-PAGE and western blotting with DV-012 to detect gp120 and pooled IgG from HIV-1-infected individuals to detect Gag proteins.

### Biosynthetic labeling and immunoprecipitation

293T cells were transfected with pΔKAD8*env* vectors. At 24-h posttransfection, the cells were incubated for 30 min in cysteine and methionine-deficient medium (MP Biomedicals), and then labeled for 45 min with 150 μCi Tran-^35^S-label (MP Biomedicals). The cells were washed and then chased in complete medium for 5 h prior to lysis. Cell lysates and clarified culture supernatants were immunoprecipitated with pooled IgG from HIV-1-infected persons and protein G Sepharose and subjected to SDS-PAGE in the presence of β-mercaptoethanol. The labeled proteins were visualized by scanning in a Fuji phosphorimager.

### Luciferase reporter assay of cell-cell fusion

Cell-cell fusion assays were conducted as previously described [27]. Briefly, 293T cells were cotransfected with pΔKAD8*env* and the bacteriophage T7 RNA polymerase expression vector, pCAG-T7 [93]. BHK21 target cells were cotransfected with pc.CCR5 (AIDS Research and Reference Reagent Program from N. Landau [94]) and pT4*luc*, a bicistronic vector that expresses human CD4 from a CMV promoter and firefly luciferase from a T7 promoter [13]. At 24 h posttransfection, targets and effectors were cocultured in triplicate in a 96-well plate (18 h, 37°C) and then assayed for luciferase activity (SteadyGlo, Promega).

### Neutralization assay

Purified IgG of brNAbs 2F5 [63], 4E10 [64] and IgGb12 [95,96] were obtained from Polymun Scientific, while the HR2 peptide analogue, C34 [43], was purchased from Genscript. Neutralization assays were conducted using TZM-bl cells (obtained from J. C. Kappes, X. Wu and Tranzyme Inc., NIH AIDS Research and Reference Reagent Program [97-99]), a HeLa cell line expressing CD4 and CCR5 and harbouring integrated copies of the luciferase and β-galactosidase genes under control of the HIV-1 promoter. Virus stocks produced by pAD8-transfected 293T cells and determined to give ~1.5 × 10^6^ relative light units (RLU) following infection of TZM-bl cells, were mixed with an equal volume of serially diluted IgG or C34 peptide and incubated for 1 h at 37°C. One hundred μl of the virus-IgG mixtures was then added to TZM-bl cells (10^4^ cells in 100 μl per well of a 96-well tissue culture plate) and incubated for 2 days prior to lysis and assay for luciferase activity (Promega, Madison, WI). For experiments with the CCR5 antagonist maraviroc (NIH AIDS Research and Reference Reagent Program [100]), the cells were preincubated for 1 h at 37°C with the drug prior to incubation with virus for 48 h. Neutralizing activities were measured in triplicate and reported as the average percent luciferase activity.

## Abbreviations

brNAb: Broadly neutralizing monoclonal antibody; C: Conserved region of gp120; DSR: Disulfide bonded region; Env: HIV-1 envelope glycoprotein; HR1: HR2, Helical regions 1 and 2 of gp41, respectively; mAb: Monoclonal antibody; MPER: Membrane-proximal ectodomain region; RT: Reverse transcriptase; V: Variable region of gp120; VSV G: Vesicular stomatitis virus glycoprotein G.

## Competing interests

The authors declare that they have no competing interests.

## Authors’ contributions

AIK, HD and PP conceived the study. AIK, AKB-M, HD and PP wrote the manuscript. AIK, AL, DNH, AKBM, HD and PP acquired experimental data and performed the data analysis. All authors read and approved the final manuscript.
